# Atrial fibrillation and its determinants after radiofrequency ablation of chronic common atrial flutter

**Published:** 2005-10-01

**Authors:** Stéphane Cade, Shahine Sedighian, Bortone Agustin, Richard Gervasoni, Christophe Jean, Florence Leclercq, Robert Grolleau, Pasquié Jean Luc

**Affiliations:** Hopital Arnaud de Villeneuve, Centre Hospitalier Universitaire, Montpellier, France

**Keywords:** atrial fibrillation, atrial flutter, follow-up, radiofrequency catheter ablation

## Abstract

**Aim:**

Atrial fibrillation (AFib) is a major clinical issue and its occurrence is the main problem after catheter ablation of atrial flutter. The long-term occurrence of AFib after common atrial flutter ablation is still matter of debate as it may influence the therapeutic approach. So, the aim of our study was to analyze the determinants and the time course of AFib after radiofrequency catheter ablation of chronic common atrial flutter.

**Methods and Result:**

89 consecutive patients (67.5 ± 12.0 yrs) underwent RF ablation of chronic common atrial flutter. 38.2 % had previous history of paroxysmal AFib. 51% had no underlying structural heart disease. Over a mean follow-up of 38 ± 13 months, the occurrence rate of AFib  progressively increased up to 32.9% at the end of follow-up. The median occurrence time for AFib was 8 months. AFib occurrence was significantly associated with previous AFib history (P=0.01) but not with the presence of underlying heart disease (P=n.s.). Of particular interest, in our study, AFib never occurred in patients without previous AFib history. Palpitations after chronic common atrial flutter ablation was mostly related to AFib.

**Conclusion:**

In conclusion, after chronic common atrial flutter ablation, AFib incidence progressively increased over the follow-up in all patients. Patients with prior AFib history appeared to be a very high risk group. In these patients, closer monitoring is mandatory and the persistent risk of AFib recurrences may justify prolonged anticoagulation policy.

## Introduction

As recently demonstrated by Natale et al catheter ablation has become first-line therapy for common atrial flutter (AFL) in patients without structural heart disease and without left atrium enlargement [1]. In a recent study by Anselme et al [2], one third of the patients still complained with palpitations after AFL ablation despite a significant improvement in quality of life and these symptoms were mostly related to atrial fibrillation (AFib). AFib is a major cause of morbidity and mortality, increasing risk for death congestive heart failure and stroke [3].  Thus, identifying patients with high risk of AFib occurrence after AFL ablation is of particularly high importance as it may influence the therapeutic approach. After AFL ablation, the long-term incidence of AFib is a major concern. However, despite numerous studies on catheter ablation of atrial flutter data are still discordant. In some studies, the risk of AFib after AFL ablation appeared to be decreased in patients with previous history of AFib [4-6] though it was not in other studies on large populations [7-9]. Moreover, some authors suggest from experimental studies an increased risk of AFib in patients with chronic atrial flutter due to more severe atrial remodeling compared to paroxysmal AFL [10].

Thus, the aim of the present study was to determine the long-term incidence of atrial fibrillation after chronic common atrial flutter ablation in a large population and to analyse its determinants.

## Methods

### Study patients and Ablation procedure

We considered 89 consecutive patients referred to our institution for chronic common AFL ablation. All patients were in chronic common AFL at the time of ablation procedure. Patients were studied in the postabsorptive state under light sedation and after all antiarrhythmic drugs except amiodarone had been stopped for > 5 half-lives. Based on the initially described technique [11], an electrophysiologically and anatomically guided ablation was performed in the IVC-TA isthmus. The aim of the ablation was termination of AFL with restoration of sinus rhythm associated to bidirectional block in the cavo-tricuspid isthmus as commonly described [12].

### Long-term follow-up

Patients were discharged the day after the procedure. Patients with previous AFib history were discharged with antiarrhythmic (AA) therapy (amiodarone 200 mg/d or flecainide 200 mg/d). All patients had oral anticoagulants for at least 6 months after the procedure. Later follow-up visits were performed by the referring cardiologist. The referring cardiologists were contacted by telephone and asked about the “arrhythmic” status of the patients. All patients were contacted by telephone to confirm their clinical status. Holter ECG recordings were systematically performed at 1 month, 3 months, 6 months and every 6 months thereafter. When a patient complained with palpitations, Holter ECG recordings were repeated until precise diagnosis was made.

### Statistical analysis

Continuous variables were expressed as mean ± SD. When pertinent, we calculated the median value as well as the 25th-75th percentile for each value to describe its distribution in the population. We defined as “Median Occurrence Time 50” the median time of AFib occurrence for 50 % of the population. The relationship between qualitative variables were assessed by Chi square-test or Fischer Exact test depending on the size of the population. Actuarial freedom from occurrence was determined using the method of Kaplan and Meier. The comparison of actuarial freedoms depending on the presence or not of one considered factor (prior history of AFib; LVEF < 50%; Chronic obstructive lung disease; AFib inducibility) was assessed using Log Rank test and Wilcoxon test. A value of P< 0.05 was considered significant.

## Results

### Study population

We studied 89 patients (79 men/ 10 women; mean age: 67.5 ± 12.0 yrs.) with chronic common isthmus-dependent AFL (Table 1). All of the patients were in chronic atrial flutter at the time of the ablation procedure. Some of them had at least 1 previous episode of atrial fibrillation before atrial flutter occurrence and were considered as pts with previous AFib. None of them had AFL induced by Ic antiarrhythmic treatment.  The median duration of chronic AFL before ablation procedure was 12 months (25th -75th percentile: 3-60 months). 38 % had previous paroxysmal AFib (median: 48 months; 25th-75th percentile: 8-72 months) but AFL always remained the major symptomatic arrhythmia. All had failed several AA drugs and 92.1 % had ongoing AA treatment including amiodarone in 71 patients. 49 % had LVEF< 50 %.  12 % had previous CABG. Chronic obstructive lung disease was observed in 20 % of the patients.

### Initial results and Follow-up

The mean follow-up duration was 38 ± 13 months. Eight patients died during follow-up (1 cerebrovascular disease, 4 coronary artery disease, 1 heart failure, 1 lung cancer, 1 acute respiratory failure in a pt with severe pulmonary disease). Two patients had dual-chamber pacemaker for sinus dysfunction and symptomatic second degree AV block 18 and 21 months respectively after ablation procedure. Thirty-four patients remained under AA therapy after the ablation procedure.

**Figure 1:**
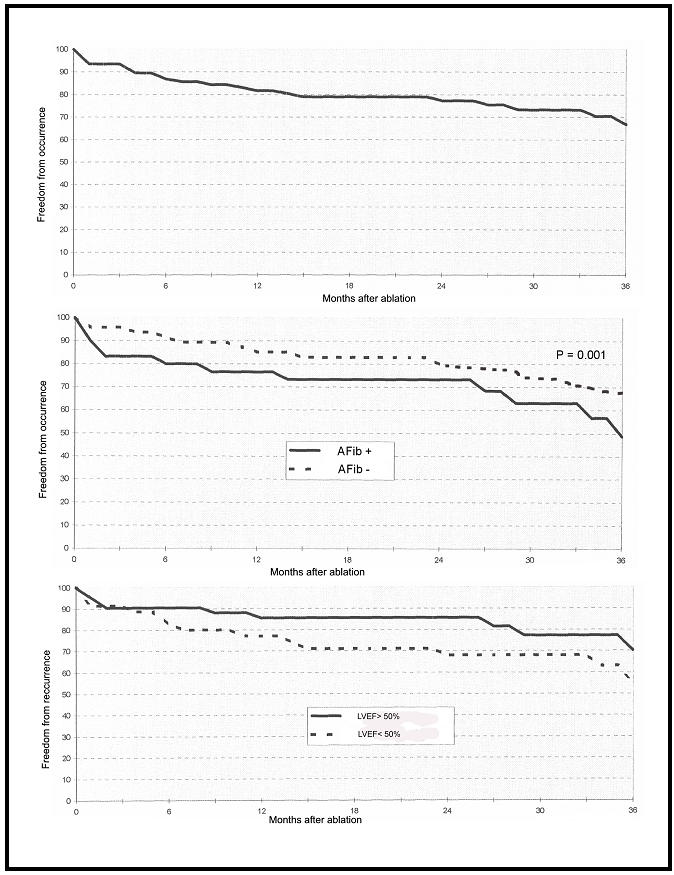
**Top**: Actuarial freedom from atrial fibrillation episodes after ablation of chronic common atrial flutter; (**Middle**): Actuarial freedom from atrial fibrillation episodes after ablation of chronic common atrial flutter considering the presence of previous history of atrial fibrillation. AFib + indicates previous history of AFib; AFib - indicates no previous history of AFib; (**Bottom**): Actuarial freedom from atrial fibrillation episodes after ablation of chronic common atrial flutter considering the LVEF < 50%.

At the end of the 3-year follow-up, 32.9 % of the patients experienced AFib (Table 2). The AFib “Median Occurrence Time 50” was 8 months (25th-75th percentile: 2-24 months, Table 2). During follow-up, 9 patients with LVEF < 50% developed chronic AFib. The actuarial freedom from AFib is represented on Figure 1. The actuarial freedom from AFib was significantly decreased when previous AFib history was present (P = 0.001; Figure 1). No significant difference in the actuarial freedom from AFib was found considering LVEF (Figure 1) and presence of chronic obstructive lung disease.

### Palpitations

Among the study population, 53 out of 89 patients visited their referring cardiologist for various symptoms. Twenty-seven patients complained with palpitations; only 4 of these patients were free of any arrhythmia. On the contrary, 5 patients had asymptomatic AFib diagnosed by Holter ECG recordings. However, palpitations were significantly associated to AFib episodes (P = 0.01).

## Discussion

In our study, after chronic common AFL ablation we observed a progressive increase in AFib incidence up to 32.9 % at 3 years. Prior AFib history was the only determinant of AFib incidence. Moreover, palpitations after AFL ablation were most of the time symptomatic of AFib episodes.

The relationships between AFL and AFib are complex. In all of the previous studies about AFL ablation [13-20] a previous AFib history was present in 20 to 55 % of the population. However, the AFib occurrence rate after AFL ablation varies widely in previously reported series. This rate was as high as 43 % as reported by Philippon et al [17], 37.5 % in the study of Saoudi et al [8] or 31 % for Hsieh et al [9]. In our study, 38.2 % of patients had previous AFib history. After AFL ablation, we had a progressive increase in AFib occurrence reaching a 32.9 %-occurrence rate by 36 months. Some authors more recently concluded that AFL ablation could reduce the incidence of AFib from 55 % to 33 % on a mean follow-up of 496 ± 335 days [6]. From our results, it appeared that the main element was time i.e. the duration of follow-up as we had a progressive increase in AFib occurrence rate. As a matter of fact, Gilligan et al [8] showed a 59 %-cumulative occurrence rate of AFib and atypical flutter at 2 years with only 27 % of patients remaining still free of atrial arrhythmias at 5 years. This may also explain a lower AFib occurrence rate of 28 % after ablation compared to a 53 %-AFib ocurrence rate before ablation found by O’Callaghan et al [16]; the 28 %-AFib occurrence rate is the 12 month-value after AFL ablation that is even higher than in our study after 1-year follow-up. Moreover, some authors found an increased incidence of early AFib after AFL ablation [20,21]. These early episodes might be transient and self-limited [19] or associated with mitral regurgitation [20]. In our study, AFib occurrence appeared progressive. These discrepancies between studies may be due to differences in the mean age of the population and more advanced heart disease. This second point might explain also the fact that patients with flutter only have a very low risk of fibrillation in our follow-up, contrary to patients with previous AFib history. Some authors have discussed a putative arrhythmogenic effect of ablation lesion [4] or would have expected an increased risk for AFib through more severe atrial remodelling during chronic AFL [10], or may be through larger anatomic conduction pathways in the chronic form [22]; in our study including only patients with chronic common flutter such an arrhythmogenic effect was not detected.

However, our study confirmed the clinical benefit of AFL ablation described by O’Callaghan et al [16] or Anselme et a [12]. Most of the patients remained asymptomatic without any AA treatment for patients without prior AFib history. Moreover, in agreement with Anselme et al [2], patients presenting with palpitations were mainly suffering from AFib. Among the possible determinants of AFib occurrence after AFL ablation, prior AFib history appeared to be a major factor. In a study by Philippon et al [17], four factors were found to predict an increased risk for developing AFib after AFL ablation: structural heart disease, previous history of AFib, high rate pacing-induced AFib and a greater number of failed AA treatments in univariate analysis. In multivariate analysis, only AFib inducibility was independently associated to late occurrence of AFib [19]. In the same manner, Da Costa et al [20] identified independent predictors of early AFib after AFL ablation: left ventricular ejection fraction and pre-ablation history of atrial fibrillation. On a longer follow-up, Hsieh et al showed that prior history of AFib and inducible AFib were independent predictors of early occurrence of AFib and that prior history of AFib was the only independent predictor of late occurrence of AFib [9]. In our study, the only factor associated to AFib occurrence was previous AFib history. As reported by Sparks et al [10], chronic atrial flutter is associated to increased early AFib inducibility but it is not demonstrated that it is associated to AFib occurrence in the long term. As opposed to previous studies [17,20,21], the presence of structural heart disease (EF<50%) was not associatedwith increased AFib occurrence in our study. These differences may be related to different stages of the arrhythmic atrial disease or of the definition and severeity of structural heart disease as suggested by Da Costa et al [20] and Philippon et al [17]. Thus, the major determinant of AFib after AFL ablation appeared to be prior AFib history. So, in patients undergoing AFL ablation, patients without prior AFib have a low risk of arrhythmia recurrence after the procedure without any AA treatment and patients with prior AFib have a high risk of arrhythmia recurrence. In these patients, AFL ablation will not relieve totally the symptoms and may necessitate complementary treatment: appropriate anticoagulation, continuation of suppressive antiarrhythmic therapy or additional AFib ablation procedure.

## Figures and Tables

**Table 1:**
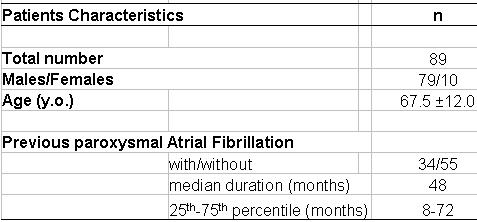
Patients characteristics

**Table 2:**
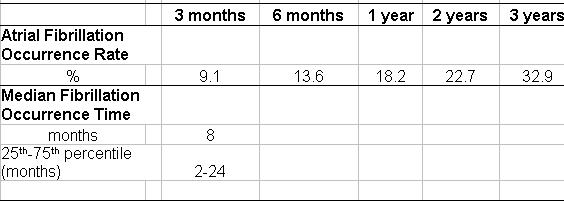
Characteristics of atrial flutter recurrence and atrial fibrillation occurrence after ablation
